# Abscisic acid metabolism to dehydrovomifoliol by *Candidatus* Liberibacter asiaticus and CLas-associated *Flavobacterium* strains CGRL1 and CGRL2

**DOI:** 10.3389/fmicb.2026.1940325

**Published:** 2026-09-15

**Authors:** Kosuke Yamada, Azusa Fukushima, Takashi Fujikawa, Hiroyuki Nakagawa, Kosuke Namba, Kazuki Fujiwara

**Affiliations:** 1 Graduate School of Agriculture, Meijo University, Nagoya, Aichi, Japan; 2 Institute for Plant Protection, National Agriculture and Food Research Organization (NARO), Tsukuba, Ibaraki, Japan; 3 Research Center for Advanced Analysis, Core Technology Research Headquarters, National Agriculture and Food Research Organization (NARO), Tsukuba, Ibaraki, Japan; 4 Department of Chemistry, Graduate School of Science, The University of Osaka, Toyonaka, Osaka, Japan

**Keywords:** abscisic acid, *Candidatus* Liberibacter asiaticus, citrus greening disease, dehydrovomifoliol, *Flavobacterium*, huanglongbing

## Abstract

**Introduction:**

Citrus huanglongbing (HLB), caused by *Candidatus* Liberibacter asiaticus (CLas), is a globally devastating plant disease characterized by severe disruption of host physiology. Although alterations in plant hormone homeostasis, including salicylic acid and abscisic acid (ABA), are hallmarks of HLB, the contribution of plant-associated microbiota to these hormonal changes remains poorly understood. Here, we investigated whether ABA depletion in HLB-affected citrus is associated with microbial metabolism.

**Methods:**

ABA and its metabolites were quantified in HLB-affected citrus tissues by liquid chromatography–mass spectrometry. The capacity of CLas strain Ishi-1 and *Flavobacterium* spp. strains CGRL1 and CGRL2 to metabolize ABA was assessed using *in vitro* culture assays. The effects of exogenous ABA on bacterial growth were also evaluated.

**Results:**

ABA concentrations were significantly decreased in symptomatic HLB-affected tissues, concomitant with the accumulation of the ABA catabolite dehydrovomifoliol. Furthermore, CLas strain Ishi-1 and *Flavobacterium* strains CGRL1 and CGRL2 degraded ABA and produced dehydrovomifoliol, whereas no such activity was detected in non-inoculated controls. Notably, exogenous ABA promoted bacterial growth, suggesting that ABA may serve as a metabolic substrate or growth-enhancing factor for these bacteria.

**Discussion:**

These findings identify microbial ABA metabolism as a potential driver of hormone depletion in HLB-affected citrus. Our results further suggest that CLas and its associated bacteria may actively reshape host hormone homeostasis through ABA metabolism, providing a mechanistic link between pathogen-associated microbial communities and hormonal imbalance during HLB development.

## 1 Introduction

Citrus huanglongbing (HLB), also known as citrus greening (CG), is one of the most destructive diseases affecting citrus production worldwide. The disease is associated with three phloem-limited bacterial species, *Candidatus* Liberibacter africanus (CLaf), *Candidatus* Liberibacter americanus (CLam), and *Candidatus* Liberibacter asiaticus (CLas). Among these, CLam and CLas are transmitted by the psyllid *Diaphorina citri*, whereas CLaf is transmitted by *Trioza erytreae* (Bové, 2006; Gottwald, 2010). CLas secretes multiple effector proteins into host tissues, contributing to characteristic symptoms such as leaf chlorosis and yellowing (Pitino et al., 2016; Clark et al., 2018; Ying et al., 2019). CLas-derived effectors elicit robust plant immune responses, including salicylic acid (SA) accumulation, callose deposition, production of reactive oxygen species (ROS), and induction of pathogenesis-related (PR) genes (Wang et al., 2017). Infected citrus exhibits pronounced hormonal imbalance, with elevated SA and altered abscisic acid (ABA) dynamics relative to healthy plants (Nehela and Killiny, 2020). These observations support a model in which HLB is an immune-mediated disease characterized by chronic activation of host defenses (Ma et al., 2022).

CLas maintains an intracellular lifestyle in both citrus hosts and psyllid vectors and possesses a highly reduced genome (~1.2 Mb), lacking many metabolic functions present in related members of the *Rhizobiaceae* (Duan et al., 2009). Despite these limitations, CLas efficiently colonizes host tissues under immune-active conditions, implying reliance on host resources and interactions with co-occurring microbial communities. Indeed, increasing evidence indicates that CLas infection reshapes the plant-associated microbiota (Blaustein et al., 2017; Yang and Ancona, 2022). Our previous work showed that members of the phylum Bacteroidetes, particularly the family *Flavobacteriaceae*, are enriched in CLas-associated microbiomes and positively correlate with proliferation of CLas Ishi-1 strain (Fujiwara et al., 2018). Furthermore, two novel *Flavobacterium* spp. strains CGRL1 and CGRL2, were recently isolated from infected citrus trees (Yamada et al., 2025). However, whether CLas-induced microbiome shifts contribute to disease development and disruption of host hormone homeostasis remains unclear.

Plant hormones are central regulators of growth, development, and responses to environmental stress. In addition to functioning as endogenous signals, they mediate interactions between plants and their associated microbiota (Adie et al., 2007; Robert-Seilaniantz et al., 2007). Plant-associated microbes can synthesize, degrade, and modulate plant hormones, thereby influencing host physiology and immune responses (Robert-Seilaniantz et al., 2011; Yang et al., 2024). Among these hormones, SA and ABA play key roles in coordinating immune responses and stress adaptation (Glazebrook, 2005; Bari and Jones, 2009; Lata and Prasad, 2011) and interact antagonistically, forming a regulatory network that balances defense activation and stress tolerance (Bari and Jones, 2009; Verma et al., 2016). Microbial metabolism of SA has been extensively characterized, with degradation occurring via the catechol (β-ketoadipate) or gentisate pathways, leading to attenuation of SA-mediated immunity and enhanced microbial colonization (Bosch et al., 2000; Zhou et al., 2001; Chowdhury et al., 2014). In contrast, microbial metabolism of ABA remains poorly understood. To date, bacterial conversion of ABA into dehydrovomifoliol has been reported only in *Rhodococcus* sp. P1Y (Yuzikhin et al., 2021), and the ecological relevance of this process in plant-associated microbiomes is largely unexplored. Whether ABA-metabolizing bacteria contribute to disease-associated hormonal imbalance or modulate immune-stress trade-offs in plant-microbe interactions remains an open question.

In this study, we investigated plant-microbiome-hormone interactions in HLB citrus, focusing on ABA metabolism. We first quantified hormone profiles in healthy and HLB citrus tissues (Figure 1A). We then examined whether CLas strain Ishi-1 and two CLas-associated *Flavobacterium* spp. strains CGRL1 and CGRL2 metabolize ABA into dehydrovomifoliol (Figures 1B,C). Through this approach, we aimed to elucidate the contribution of pathogen-associated microbial communities to host hormone imbalance during HLB pathogenesis.

**Figure 1:**
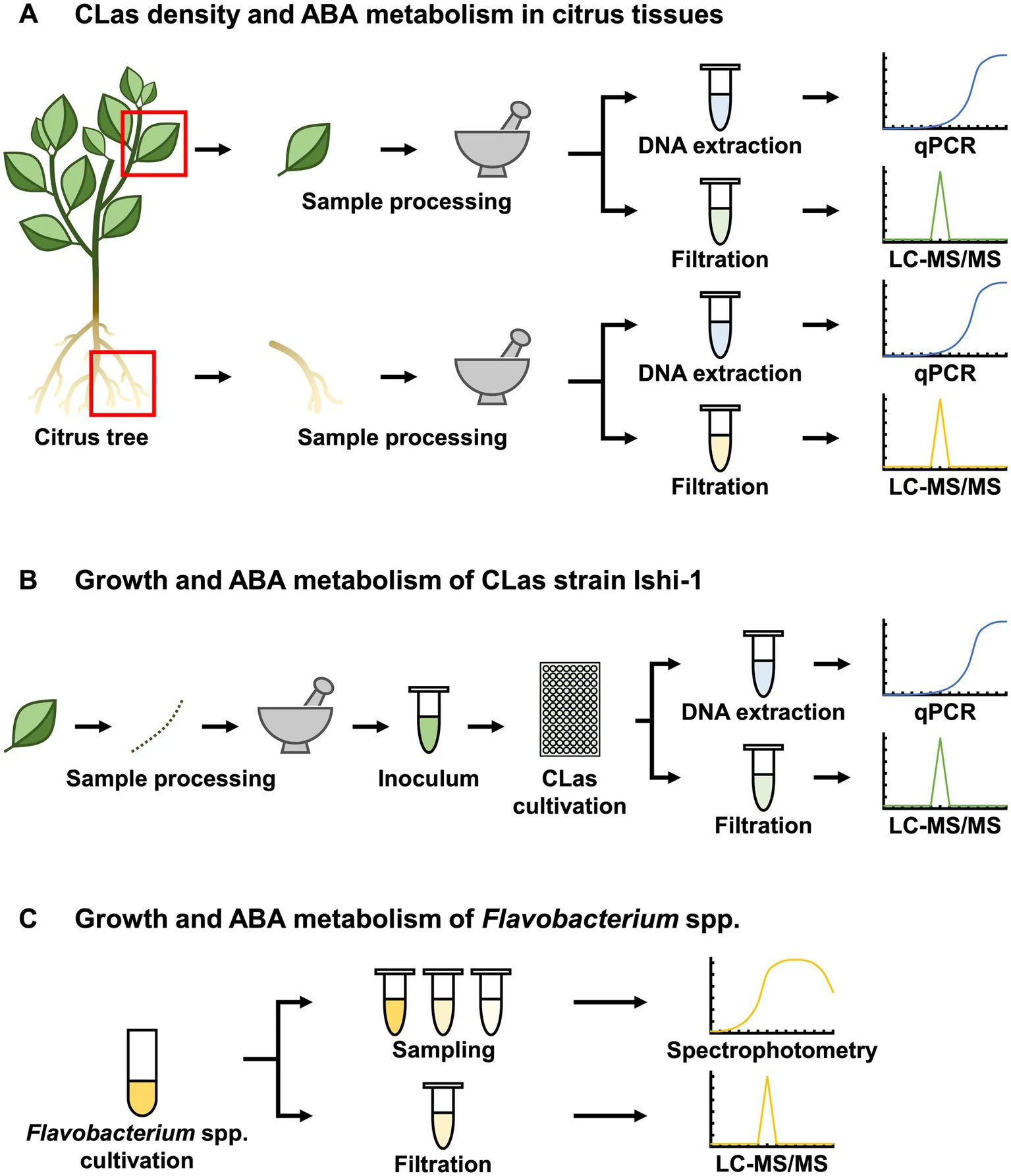
Experimental workflow for quantifying bacterial density and ABA metabolism in citrus tissues and bacterial cultures. **(A)** Healthy and HLB citrus leaf and root tissues were analyzed to quantify CLas density by qPCR and ABA and dehydrovomifoliol levels by LC-MS/MS. **(B)** CLas strain Ishi-1 was cultured in ABA-supplemented medium to evaluate the effect of ABA on bacterial growth and metabolism. Bacterial density was quantified by qPCR, whereas ABA and dehydrovomifoliol were quantified by LC-MS/MS. **(C)** *Flavobacterium* spp. strains CGRL1 and CGRL2 were cultured in ABA-supplemented medium to assess bacterial growth and ABA metabolism. Bacterial growth was quantified spectrophotometrically, and ABA and dehydrovomifoliol were quantified by LC-MS/MS.

## 2 Materials and methods

### 2.1 Plant growth and experimental setup

Two-year-old rough lemon (*Citrus jambhiri* Lush.) trees were graft-inoculated with CLas strain Ishi-1 and maintained in a quarantined phytotron. Inoculated citrus plants were grown at 25 °C ± 5 °C under natural photoperiod conditions. Plants were fertilized with HYPONeX powdered fertilizer (Hyponex JAPAN, Osaka, Japan) once every 2 weeks and Eido Ball Ca (KINCHO garden products, Tokyo, Japan) once a month throughout the experimental period. HLB infection was confirmed 1 year after inoculation based on the development of characteristic foliar symptoms and PCR detection of CLas, as previously described (Fujikawa et al., 2013). Healthy leaves were collected from non-inoculated, PCR-negative trees. Asymptomatic leaves were collected from CLas-positive trees that showed no visible HLB-associated symptoms but were PCR-positive for CLas at the time of sampling. Symptomatic leaves were collected from CLas-positive trees exhibiting characteristic HLB-associated foliar symptoms and confirmed to be PCR-positive for CLas. Among the 3-year-old trees confirmed to be HLB infected, eight trees were randomly selected for subsequent experiments. To minimize variation associated with leaf developmental stage, leaves at the third to eighth positions from the shoot apex were selected for all groups.

### 2.2 Bacterial cultivation

CLas strain Ishi-1 inoculum was prepared following the method described by Fujiwara et al. (2018). Briefly, CLas-infeced leaves were surface sterilized with 70% ethanol. Approximately 50 mg of leaf-midribs was cut, finely chopped, and homogenized in 400 μL sterilized distilled water in a BioMasher III mini-homogenized tube (Nippi, Tokyo, Japan). Then, the samples were centrifuged for 2 min at 7,000 × g. After centrifugation, 400 μL of flow-through solution was discarded and the precipitate was resuspended in 400 μL of sterilized distilled water. These suspensions were used as the culture inoculum. Strain Ishi-1 was cultured in 200 μL of CG liquid medium (Fujiwara et al., 2018) that adjusted to a final concentration of 10 μM of (*Z*)-ABA, 10 μM of (*E*)-ABA, and untreated medium. Cultures were prepared in 96 well plates using 10 μL of inoculum and incubated at 25 °C with shaking at 160 rpm for 14 days. CLas strain Ishi-1 was quantified by qPCR using the same method as described below.

Bacterial strains, *Flavobacterium* spp. strains CGRL1 and CGRL2, were pre-cultured in 5 mL of CG liquid medium and in test tubes at 25 °C with shaking at 140 rpm for 3 days, followed by incubation in 5 mL of modified mineral salt medium supplemented with glucose (Supplementary Table S2) under the same conditions for 7 days. The resulting pre-cultures were adjusted to an optical density at 600 nm (OD_600_) of 0.100 (3.3 × 10^7^ CFU/mL), measured using the Ultrospec 3,100 pro UV/Visible Spectrophotometer (Amersham Biosciences, Little Chalfont, UK), and used to inoculate the main cultures. For the main cultures, 5 mL of CG liquid medium supplemented with 10 μM of (*Z*)-ABA, 10 μM of (*E*)-ABA at the final concentration, as well as untreated CG liquid medium, were incubated in test tubes at 25 °C with shaking at 140 rpm for 7 days. Similarly, 5 mL of mineral salt medium supplemented a final concentration of 10 μM of (*Z*)-ABA, 10 μM of (*E*)-ABA, and untreated mineral salt medium were incubated under the same condition for 10 days. Bacterial growth assessed by measuring the optical density at an OD_600_ at 2-days intervals using the Ultrospec 3,100 pro UV/Visible Spectrophotometer.

### 2.3 CLas quantification

The healthy and HLB citrus leaves and roots were surface sterilized with 70% ethanol. For leaf samples, approximately 50 mg of midrib from a leaf was excised and homogenized using a mortar and pestle. For root samples, approximately 50 mg of root tissue was homogenized using a mortar and pestle. Total DNA was extracted from the homogenized samples using the DNeasy Plant Mini Kit (Qiagen, Hilden, Germany), and CLas cell concentrations were determined by quantitative PCR (qPCR) using the primer set Las931/LSS (Fujiwara et al., 2018). The qPCR reactions were carried out with SYBR Premix Ex Taq II (Tli RNase H Plus; Takara, Shiga, Japan) according to the manufacturer’s instructions with minor modifications. Thermal cycling consists of an initial denaturation at 96 °C for 10 min, followed by 45 cycles of 96 °C for 30 s, 55 °C for 30 s, and 72 °C for 30 s. Melting curve analysis was performed with 96 °C for 15 s, 55 °C for 1 min, and a gradual increase from 55 °C to 96 °C. CLas cell concentrations were calculated based on the relationship between DNA quantity and the molarity of CLas 16S rDNA (Degen et al., 2006). Template DNA was adjusted to 5.0 ng/μL, and a 10-fold serial dilution series was used to generate a standard curve.

### 2.4 ABA and dehydrovomofoliol quantification

Leaf and root tissues were surface-sterilized with 70% ethanol. A total of 200 mg of tissue from healthy and HLB citrus trees was homogenized in 1.0 mL of Milli-Q water using a mortar and pestle. The homogenates were centrifuged at 12,000 rpm for 5 min, and the resulting supernatants were filtered through a 0.2 μm hydrophilic Minisart RC syringe filter (Sartorius, Göttingen, Germany) using a 1.0 mL tuberculin syringe with a slip tip (Terumo, Tokyo, Japan). The filtered extracts were immediately frozen in liquid nitrogen and stored at −80 °C.

For quantification of changes in ABA and dehydrovomifoliol production by bacteria, culture supernatants of CLas strain Ishi-1 were collected at 0 and 1 month, and those of *Flavobacterium* spp. strains CGRL1 and CGRL2 were collected at 0 and 7 days of the main cultures. Supernatants were obtained by filtration through a 0.2 μm filter using a 1.0 mL syringe. The filtrates were immediately frozen in liquid nitrogen and stored at −80 °C. ABA and dehydrovomifoliol were subsequently quantified by LC-MS/MS under the conditions described below.

### 2.5 LC-MS analysis conditions

Synthesis of (*Z*)-ABA, (*E*)-ABA, and dehydrovomifoliol (Figure 2) were carried out according to the methods reported by Acemoglu et al. (1988) and Robert et al. (1968). Detection and quantification were performed with a 4,000 QTRAP LC-MS/MS system (Sciex, CA, USA) equipped with an atmospheric pressure electro-spray ionization (ESI) source (turbo spray) and 1,290 Series HPLC system (Agilent Technologies, Waldbronn, Germany). Data was acquired with injection volume of 3 μL using the Analyst 1.6.2 software (Sciex) with the multiple reaction monitoring (MRM) algorithm. For ABA and dehydrovomifoliol, ionization was conducted with an ESI probe in negative polarity in the following settings: source temperature 400 °C, curtain gas 10 psi, ion source gas 1 (sheath gas) 70 psi, ion source gas 2 (drying gas) 80 psi, and ion spray voltage −4,500 V. Optimization of the analyte-dependent MS/MS parameters was performed by direct infusion of standard solutions. MS and MS/MS parameters (such as the selection of the most abundant MRM transitions, declustering potentials, collision energies, cell exit potentials) were optimized for all analytes (Figure 2, Supplementary Table S1). Chromatographic separation was performed using a TSK-GEL ODS-100 V column (150 mm × 2.0 mm i.d., 5 μm particle size) (TOSOH, P/N 0021458), maintained at 40 °C with the carrier solvent composed of 10 mM ammonium acetate aqueous solution/acetic acid (99.9:0.1, v/v; eluent A) and acetonitrile/acetic acid (99.9:0.1, v/v; eluent B). Both solvents were prepared with LC-MS grade (water, acetonitrile) or pure grade (acetic acid) chemicals. The gradient program was as follows: 5% B (0–1 min), 5% B to 70% B (1–11 min), 70% B to 95% B (11–12 min), held at 95% B (12–14 min), reduced to 5% B (14–14.1 min), and held at 5% B (14.1–18 min). The flow rate was set to 0.2 mL/min.

**Figure 2:**
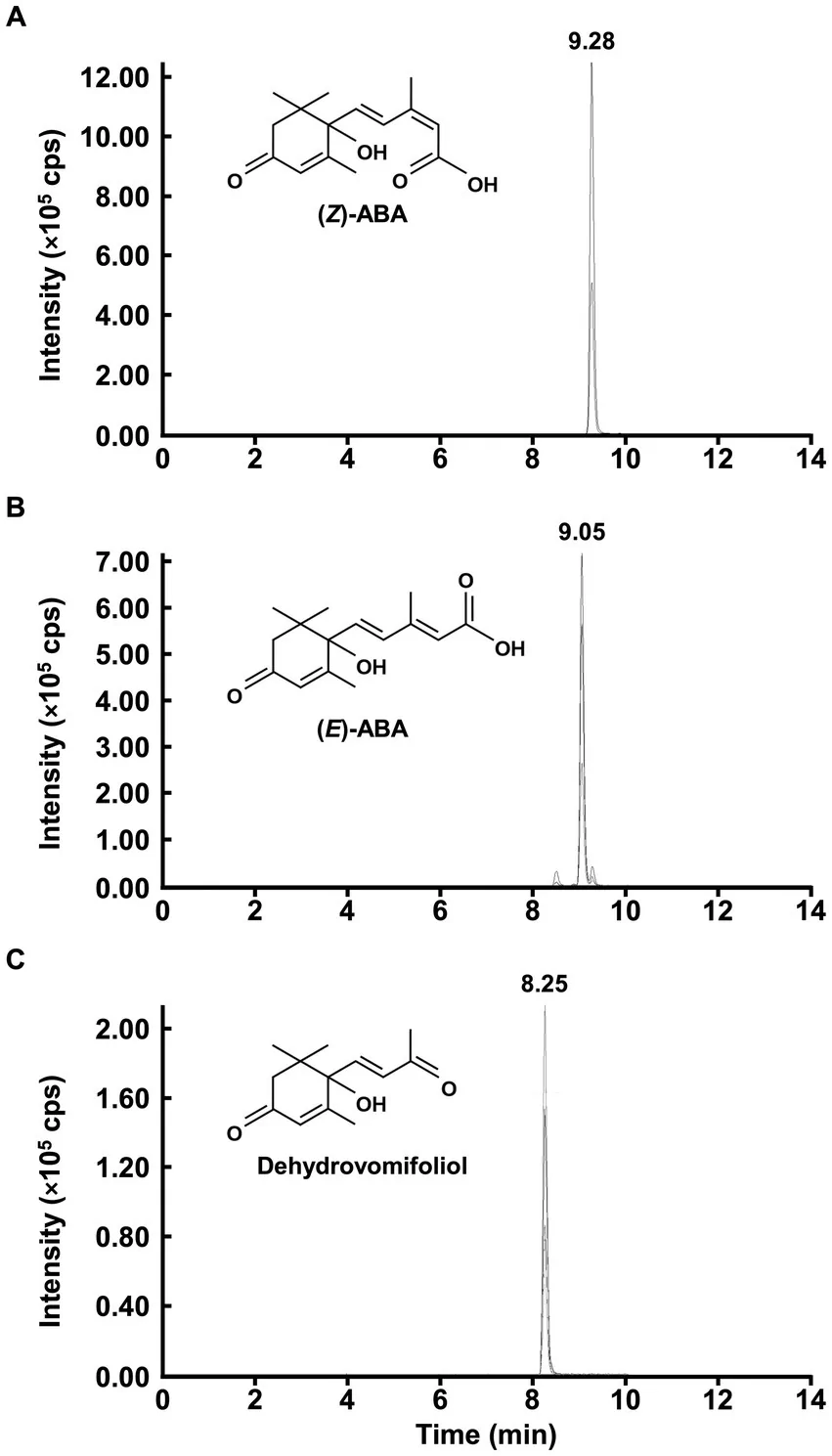
Representative LC-MS/MS chromatograms of ABA and dehydrovomifoliol standards. Multiple reaction monitoring (MRM) chromatograms of authentic standards of **(A)** (*Z*)-ABA, **(B)** (*E*)-ABA, and **(C)** dehydrovomifoliol are shown. Standard solutions were prepared at 1,000 ng/mL, and 3 μL of each solution was injected. The values above the chromatographic peaks indicate the retention times in minutes. LC-MS/MS analysis was performed under the conditions described in the Materials and Methods.

## 3 Results

### 3.1 Symptomatic infection with CLas disrupts plant hormone balance

We characterized healhy (Figure 3A) and HLB citrus leaves (Figures 3B, C) and investigated whether CLas density differed between asymptomatic and symptomatic development using qPCR analysis. CLas was detected in both asymptomatic and symptomatic leaves, with no significant difference in bacterial density between asymptomatic and symptomatic leaves (Figure 3D; *p* < 0.35). The CLas densities were 2.98 × 10^7^ (±0.71 × 10^7^ SD) cells/g FW in asymptomatic leaves and 3.52 × 10^6^ (±0.75 × 10^7^ SD) cells/mL in symptomatic leaves. Also, in symptomatic roots, CLas density was 3.05 × 10^6^ (±0.52 × 10^6^ SD) cells/g FW (Figure 3E).

**Figure 3:**
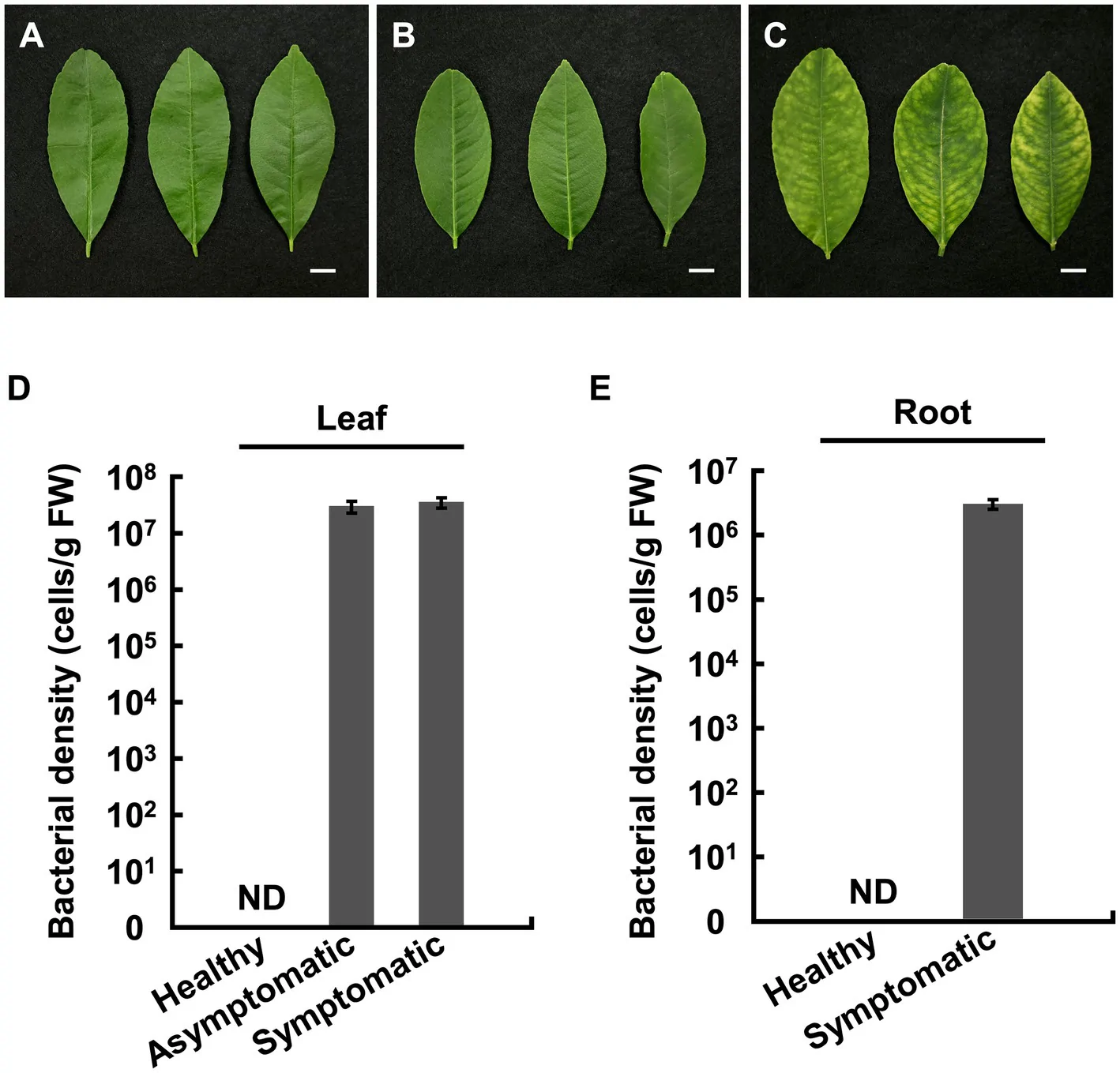
CLas density in citrus leaves and roots. Representative images of healthy **(A)**, asymptomatic **(B)**, and symptomatic **(C)** citrus leaves. Asymptomatic and symptomatic leaves were collected from HLB citrus tree. Scale bars, 1 cm. CLas density in citrus leaves **(D)** and roots **(E)** were quantified by qPCR. Statistical significance was determined using Welch’s *t*-test (*p* < 0.05). ND, not detected. Data are presented as mean ± SD (*n* = 15).

LC-MS/MS analysis revealed that CLas infection altered the plant hormone homeostasis in citrus tissues. ABA concentrations were significantly lower in symptomatic HLB leaves and roots than in healthy tissues, whereas dehydrovomifoliol concentrations were significantly higher (*p* < 0.01). In symptomatic tissues, ABA levels were 13.11 (±2.15 SD) ng/g FW in leaves and 5.24 (±0.39 SD) ng/g FW in roots, compared with 63.98 (±18.86 SD) ng/g FW and 12.93 (±3.60 SD) ng/g FW, respectively, in healthy tissues (Figures 4A,B). In contrast, dehydrovomifoliol levels in symptomatic tissues were 13.30 (±6.18 SD) ng/g FW in leaves and 64.04 (±12.04 SD) ng/g FW in roots, compared with 3.49 (±2.21 SD) ng/g FW and 24.47 (±10.13 SD) ng/g FW, respectively, in healthy tissues (Figures 4C,D). In asymptomatic HLB leaves, ABA concentrations tended to be lower and dehydrovomifoliol concentrations higher than in healthy leaves (Figures 4A,C); however, these differences were not statistically significant (*p* < 0.96 in ABA, *p* < 0.40 in dehydrovomiforiol). ABA and dehydrovomifoliol levels in asymptomatic leaves were 59.32 (±11.88 SD) ng/g FW and 7.61 (±4.47 SD) ng/g FW, respectively, indicating that dehydrovomifoliol accumulation is induced in response to CLas infection.

**Figure 4:**
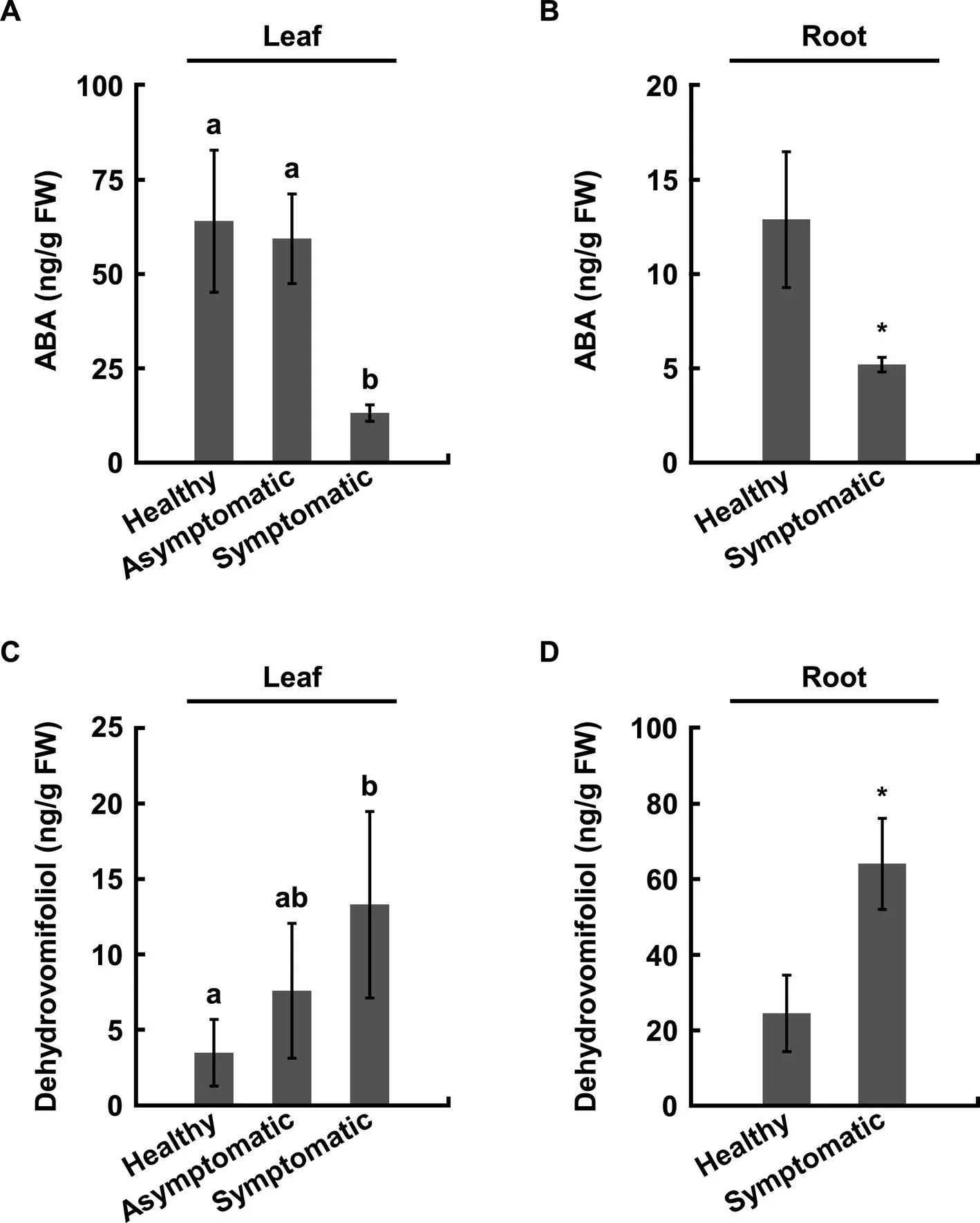
ABA and the ABA metabolite dehydrovomifoliol concentrations in healthy and HLB citrus leaves and roots. ABA concentrations in these citrus leaves **(A)** and roots **(B)**. Dehydrovomifoliol concentrations in these citrus leaves **(C)** and roots **(D)**. Statistical significance in leaves and roots was determined using the Steel-Dwass test (*p* < 0.01) and Welch’s *t* test (*p* < 0.01), respectively. Data are presented as means ± *SD* (*n* = 12).

### 3.2 Decreases in ABA are induced by bacteria *in vitro*

Inoculation with CLas strain Ishi-1 or *Flavobacterium* spp. strains CGRL1 and CGRL2 resulted in a reduction of ABA *in vitro*. The relative ABA concentration decreased significantly to 10.5% (±0.7 SD) in (*Z*)-ABA-supplemented CG liquid medium and to 41.0% (±1.3 SD) in (*E*)-ABA-supplemented CG liquid medium after incubation with strain Ishi-1 (Figure 5A; *p* < 0.01). In contrast, no significant changes were observed in the corresponding non-inoculated controls (Figure 5A; *p* < 0.86 in (*Z*)-ABA-supplemented medium, *p* < 0.48 in (*E*)-ABA-supplemented medium). Similarly, inoculation with strain CGRL1 or CGRL2 in CG liquid medium supplemented with either (*Z*)-ABA or (*E*)-ABA resulted in significant decreases in ABA concentration after cultivation (Figure 5B; *p* < 0.05). In cultures inoculated with strain CGRL1, ABA levels decreased by 82.6% (±8.6 SD) and 74.8% (±14.8 SD) under (*Z*)-ABA and (*E*)-ABA supplementation, respectively. For strain CGRL2, ABA concentrations were reduced by 21.9% (±1.1 SD) and 23.1% (±1.9 SD) under the same conditions. No significant changes were detected in the corresponding non-inoculated medium (Figure 5B; *p* < 0.95 in (*Z*)-ABA-supplemented medium, *p* < 0.66 in (*E*)-ABA-supplemented medium). These results indicate that these bacterial strains have the capacity to degrade ABA.

**Figure 5:**
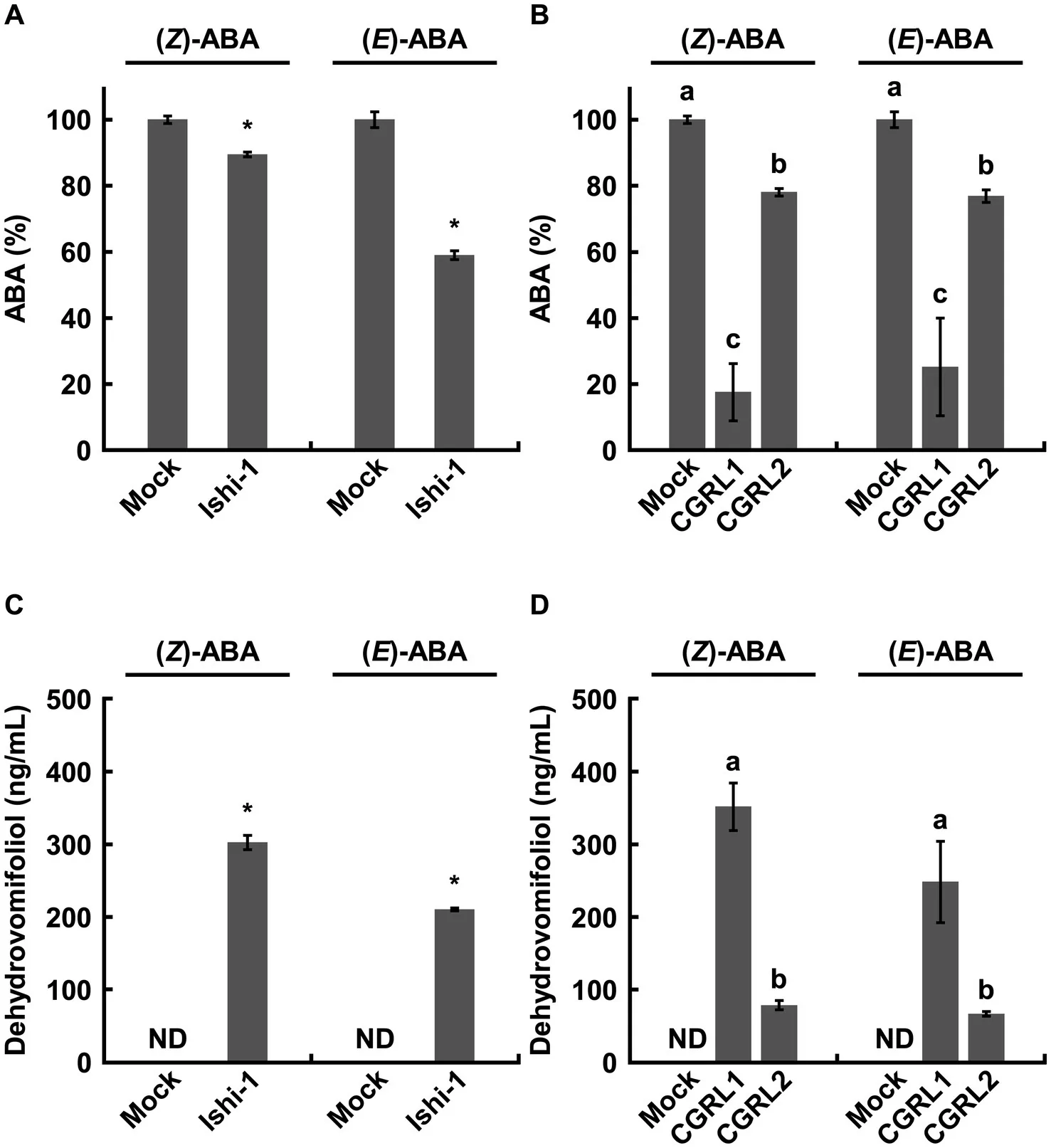
Alteration of ABA metabolism by CLas strain Ishi-1 and CLas-associated *Flavobacterium* spp. strains CGRL1 and CGRL2 during *in vitro* cultivation. Relative ABA concentrations for CLas strain Ishi-1 **(A)** and *Flavobacterium* spp. strains **(B)**. Dehydrovomifoliol concentrations in the culture medium of CLas strain Ishi-1 **(C)** and *Flavobacterium* spp. strains **(D)**. Concentrations were determined in culture medium supplemented with 10 μM (*Z*)-ABA or **(*E*)**-ABA using filtered supernatants collected after 1 month of cultivation for CLas strain Ishi-1 and after 7 days for *Flavobacterium* spp. strains. Statistical significance was assessed using the Welch’s *t* test (*p* < 0.01) for CLas strain Ishi-1 and Steel-Dwass test (*p* < 0.05 in relative ABA concentrations, *p* < 0.01 in dehydrovomifoliol concentrations) for *Flavobacterium* spp. strains. ND, not detected. Data are presented as means ± SD (*n* = 5).

### 3.3 Bacteria induce dehydrovomifoliol production *in vitro*

A significant accumulation of dehydrovomifoliol was observed following inoculation with CLas strain Ishi-1 or *Flavobacterium* spp. strains CGRL1 and CGRL2 *in vitro* (*p* < 0.01). In CG liquid medium supplemented with either (*Z*)-ABA or (*E*)-ABA, inoculation with strain Ishi-1 resulted in the production of dehydrovomifoliol at concentrations of 302.58 (±9.74 SD) ng/mL and 210.60 (±1.89 SD) ng/mL, respectively (Figure 5C). Similarly, in CG liquid medium inoculated with strain CGRL1, dehydrovomifoliol concentrations reached 351.78 (±32.63 SD) ng/mL and 248.35 (±55.94 SD) ng/mL under (*Z*)-ABA and (*E*)-ABA treatments, respectively (Figure 5D). Consistent with this trend, cultures inoculated with strain CGRL2 produced 78.71 (±6.35 SD) ng/mL and 66.89 (±3.25 SD) ng/mL under (*Z*)-ABA and (*E*)-ABA conditions, respectively (Figure 5D). In contrast, dehydrovomifoliol was not detected in the corresponding non-inoculated medium (Figures 5C,D). These findings suggest that these bacterial strains are capable of converting ABA into dehydrovomifoliol *in vitro*.

### 3.4 ABA treatment enhances bacterial growth

We investigated the effects of ABA on the growth of CLas strain Ishi-1 and *Flavobacterium* sp. strain CGRL1 *in vitro*. ABA supplementation enhanced the growth of strain Ishi-1 and transiently increased the growth of strain CGRL1 relative to non-supplemented controls (Figure 6). For strain Ishi-1, cell density increased from 628.34 (±66.57 SD) cells/mL at inoculation to 1022.79 (±55.23 SD) cells/mL in control medium, to 1692.17 (±278.93 SD) cells/mL in (*Z*)-ABA-supplemented medium, and to 1818.93 (±327.38 SD) cells/mL in (*E*)-ABA-supplemented medium, with significantly higher densities in the presence of (*Z*)- and (*E*)-ABA (*p* < 0.01). For strain CGRL1, no differences in growth were observed among treatments up to 4 days after inoculation (*p* < 0.30). At 6 days, however, cell densities reached 9.43 × 10^7^ (±3.60 × 10^4^ SD) CFU/mL and 8.90 × 10^6^ (±4.91 × 10^4^ SD) CFU/mL in (*Z*)-ABA- and (*E*)-ABA-supplemented medium, respectively, both of which were significantly higher than in the control medium (7.38 × 10^6^ ± 7.50 × 10^4^ SD CFU/mL; *p* < 0.01). Thereafter, cell densities gradually converged across treatments. These results indicate that ABA promotes the growth of strain Ishi-1 and strain CGRL1, suggesting that ABA may serve as a nutrient source or growth-promoting factor.

**Figure 6:**
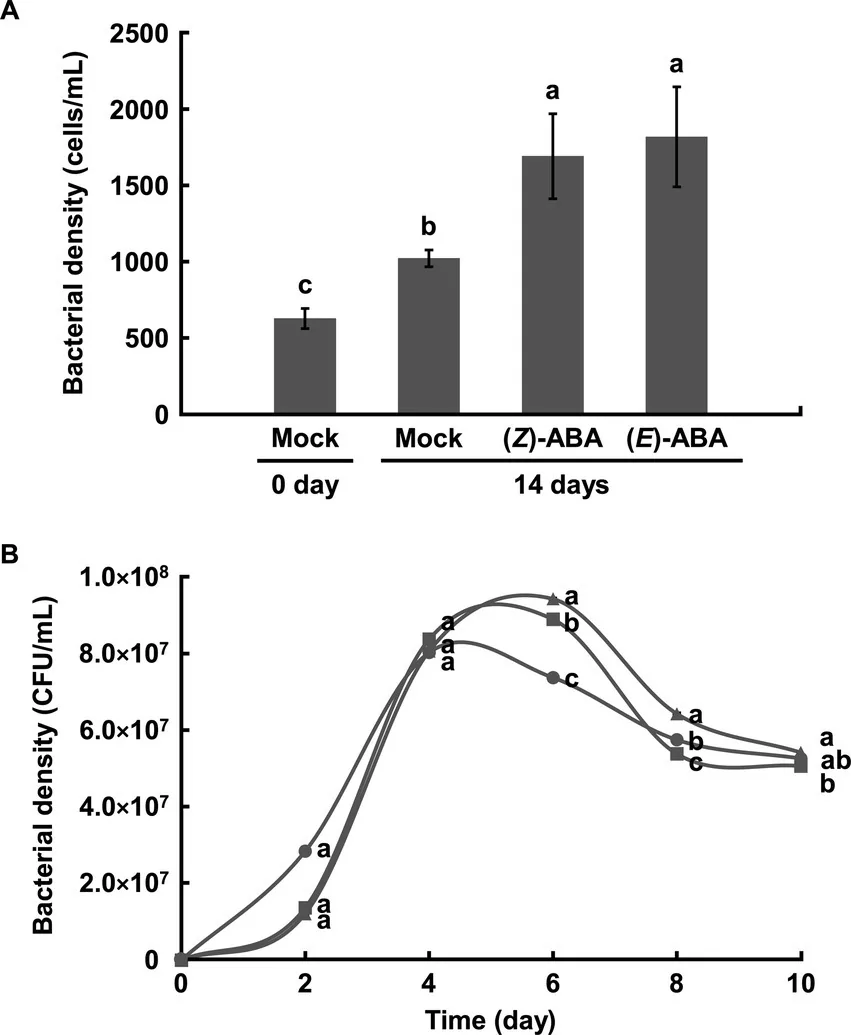
Growth of CLas strain Ishi-1 and CLas-associated *Flavobacterium* sp. strain CGRL1 under ABA supplementation. Growth of **(A)** CLas strain Ishi-1 and **(B)** *Flavobacterium* sp. strain CGRL1 under ABA supplementation. Density of CLas strain Ishi-1 was quantified by qPCR using DNA extracted from cultures grown for 14 days in medium supplemented with 10 μM (*Z*)-ABA or (*E*)-ABA and non-supplemented. Growth of *Flavobacterium* sp. strain CGRL1 was monitored every 2 days by measuring optical density at 600 nm (OD600) in cultures supplemented with 10 μM (*Z*)-ABA (triangle) or (*E*)-ABA (square) and non-supplemented (circle). Statistical significance was assessed using Steel-Dwass test (*p* < 0.01) for CLas strain Ishi-1 and Tukey’s HSD test (*p* < 0.01) for *Flavobacterium* spp. strains. Data are presented as means ± SD (*n* = 15 for CLas strain Ishi-1, *n* = 3 for *Flavobacterium* sp. strain CGRL1).

## 4 Discussion

Plant hormones regulate plant responses to biotic and abiotic stresses (Robert-Seilaniantz et al., 2007). ABA primarily mediates abiotic stress responses such as drought, whereas SA is central to defense against biotic stress, including pathogen attack (Loake and Grant, 2007; Bari and Jones, 2009; Lata and Prasad, 2011). These signaling pathways often act antagonistically and form a complex regulatory network that modulates plant stress responses (Bari and Jones, 2009; Verma et al., 2016). In addition to plant-derived regulation, microorganisms can modulate host hormone homeostasis by degrading phytohormones, such as the conversion of SA to catechol and ABA to dehydrovomifoliol, thereby attenuating defense signaling and facilitating colonization (Bosch et al., 2000; Zhou et al., 2001; Chowdhury et al., 2014; Yuzikhin et al., 2021). In HLB citrus trees, altered hormonal profiles, including increased SA and decreased ABA levels, have been reported (Nehela and Killiny, 2020). We therefore hypothesized that these imbalances arise not only from host responses but also from microbial activity associated with CLas infection. Consistent with this hypothesis, we observed a significant accumulation of dehydrovomifoliol in HLB citrus tissues. Moreover, CLas strain Ishi-1 and CLas-associated *Flavobacterium* spp. strains CGRL1 and CGRL2 degraded ABA and produced dehydrovomifoliol *in vitro*. Together, these findings suggest that CLas and its associated microbiota contribute, at least in part, to the disruption of host hormone homeostasis during infection.

A central objective of this study was to determine how ABA influences the growth of CLas and associated bacteria. We found that ABA supplementation promoted the growth of strain Ishi-1 and sustained the growth of strain CGRL1. In addition, these strains converted ABA into dehydrovomifoliol. Although the metabolic pathway underlying this conversion remains unresolved, structural comparison suggests that dehydrovomifoliol may be derived from ABA through cleavage of the C-4′ and C-5′ double bond and subsequent oxidation of the carboxyl group to a carbonyl group, resulting in a shortened side chain. This transformation implies the involvement of decarboxylase-like or oxidative enzymes encoded by CLas and *Flavobacterium* spp. (Figure 7). Transcriptome data from ABA-degrading bacteria suggest that growth in ABA-supplemented medium enhances the expression of genes related to fatty acid metabolism, and that enzymes involved in this process may participate in ABA degradation (Gogoleva et al., 2020; Yuzikhin et al., 2022). Moreover, fatty acid metabolism is altered in HLB citrus trees (Suh et al., 2018). These observations suggest that CLas infection-associated changes in lipid metabolism, possibly including bacterial fatty acid metabolism, may contribute, at least in part, to the metabolic imbalance associated with HLB symptom development.

**Figure 7:**
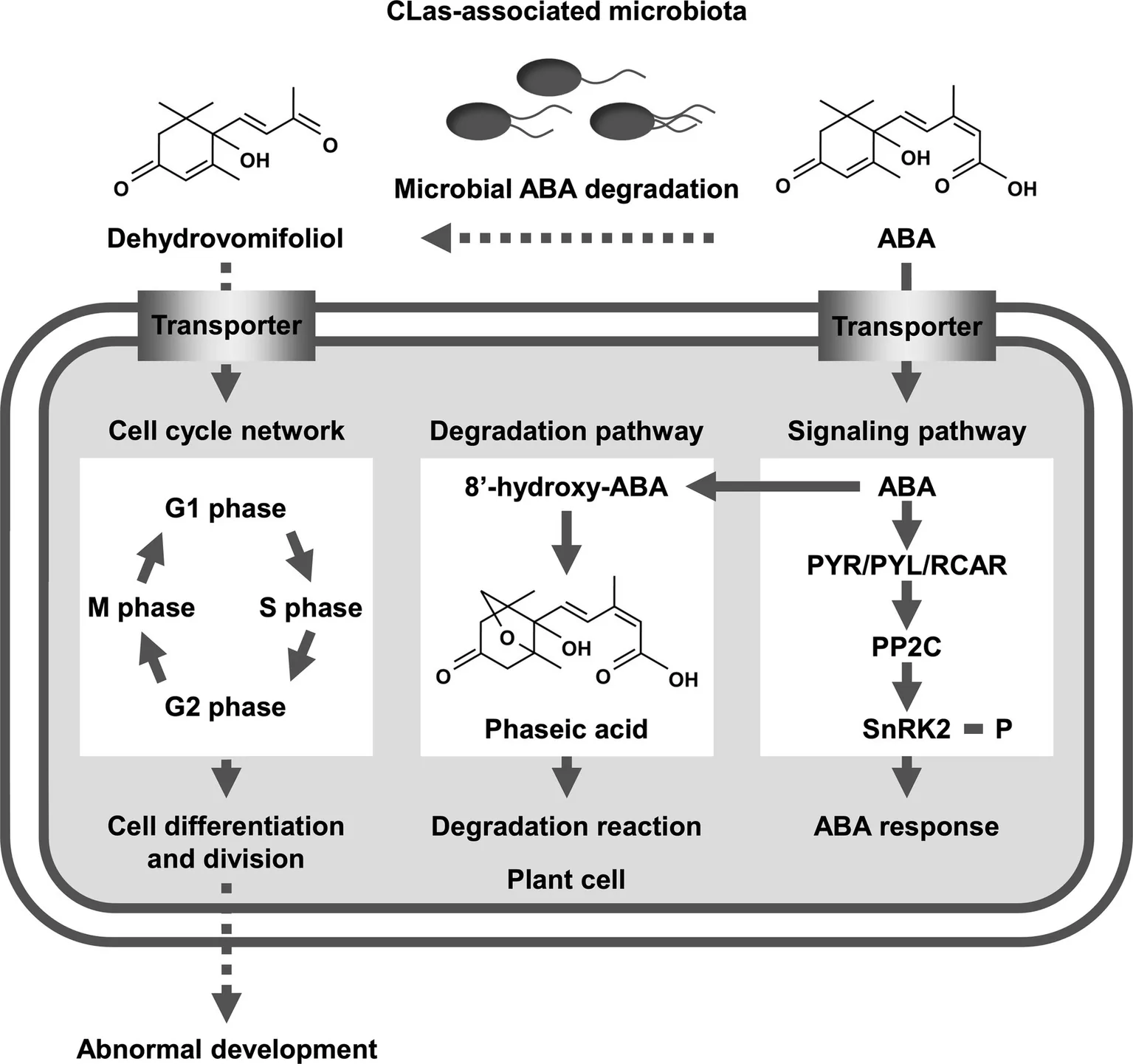
Hypothetical model illustrating interactions among ABA and CLas-associated microbiota in HLB citrus. ABA is taken up into plant cells via transporters, activates ABA-responsive signaling pathways, and is metabolized through a plant catabolic pathway involving 8′-hydroxy-ABA and phaseic acid. We hypothesize that CLas strain Ishi-1 and CLas-associated microbiota utilize ABA as a substrate and convert it into dehydrovomifoliol via a bacterial degradation pathway. This microbially derived metabolite accumulates in HLB citrus tissues and, as a phytotoxic compound, may interfere with cellular processes, including cell cycle regulation, contributing to disease symptoms such as abnormal development of leaves and fruits.

CLas infection is associated with pronounced morphological abnormalities in citrus, including leaf deformation and fruit malformation (Bové, 2006). Our data show that dehydrovomifoliol accumulates to significantly higher levels in infected tissues. Notably, exogenous dehydrovomifoliol has been reported to inhibit root elongation and leaf growth in Italian ryegrass (*Lolium multiflorum* Lam.) and cress (*Lepidium sativum* L.), indicating phytotoxic activity (Kyaw et al., 2022). These observations raise the possibility that dehydrovomifoliol contributes to disease-associated developmental defects. However, the molecular mechanisms underlying its effects on plant cells remain largely unknown and warrant further investigation. Insights into possible mechanisms can be drawn from studies in animal systems, where dehydrovomifoliol exhibits diverse bioactivities (Gyi et al., 2025; Ma et al., 2022; Xi et al., 2021). This compound, commonly found in medicinal plants used in traditional herbal medicine, has been reported to exert antiproliferative effects by downregulating E2F transcription factor 1, a key regulator of the cell cycle, and to modulate lipid metabolism in animal cells (Denechaud et al., 2016; Xi et al., 2021). In the present study, the accumulation of dehydrovomifoliol in HLB citrus tissues, together with its potential microbial origin, suggests that pathogen-associated metabolic processes generate bioactive compounds within HLB development. These compounds may disrupt cell cycle regulation, thereby contributing to abnormal development in citrus tissues, including immature development and asymmetric tissue development (Bové, 2006; Kyaw et al., 2022).

Possible reasons for bypassing the plant ABA degradation pathway in infected citrus may extend beyond the simple degradation or utilization of ABA and instead reflect a broader reorganization of host-microbe metabolism during HLB development. In healthy plants, ABA homeostasis is regulated through biosynthesis, catabolism, and conjugation, with CYP707A-mediated 8′-hydroxylation representing the major catabolic route and leading to the formation of phaseic acid (PA) (Mo et al., 2024; Figure 7). Thus, PA represents a major product of plant ABA catabolism. By contrast, bacteria can metabolize ABA through distinct catabolic routes. For example, *Rhodococcus* sp. P1Y utilizes ABA as a carbon source and produces dehydrovomifoliol as an intermediate of bacterial ABA degradation (Yuzikhin et al., 2021, 2022). Our findings that CLas and CLas-associated *Flavobacterium* strains convert ABA to dehydrovomifoliol, together with its accumulation in HLB citrus, suggest that microbial metabolism provides an alternative route for ABA turnover in infected citrus. The biological significance of this alternative route may lie not simply in ABA removal, but in the conversion of a host signaling molecule into a chemically distinct microbial metabolite. Recent work has highlighted the importance of bidirectional chemical dialogs between hosts and microorganisms, in which host-derived metabolites influence microbial physiology while microbial metabolism reciprocally modifies the chemical environment of the host (Diab et al., 2026). From this perspective, ABA may function not only as a phytohormone but also as a metabolic substrate at the host-microbe interface. Its conversion to dehydrovomifoliol could therefore represent a form of metabolic rewiring through which microbial activity alters the fate and signaling dynamics of a host hormone while generating a distinct chemical signal. Thus, pathogen-associated microbial metabolism may contribute to the rewiring of host hormone homeostasis rather than merely responding to infection-induced changes in hormone abundance. The low-level presence of dehydrovomifoliol in healthy citrus further suggests that this metabolic interaction may be part of a normal host-microbiome chemical network that becomes dysregulated during HLB development, rather than being intrinsically pathological. Although its origin in healthy tissues remains unresolved, its basal occurrence may reflect a constitutive chemical network linking citrus plants with its resident microbiota. Plant metabolites and phytohormones can shape microbial community assembly and activity, while microbial metabolites reciprocally influence host physiology and immunity (Sánchez-Cañizares et al., 2017; Spooren et al., 2024). Thus, the distinction between healthy and HLB-affected citrus may not be the presence or absence of microbial ABA metabolism, but rather a change in its magnitude, context, and integration within the host-microbiome metabolic network.

CLas infection may disrupt this metabolic equilibrium by altering both host hormone homeostasis and microbiome composition. Changes in the microbial community could modify the capacity for host-derived metabolite transformation, thereby increasing the flux of ABA through microbial rather than plant catabolic pathways and promoting dehydrovomifoliol accumulation. This process could establish a feedback loop in which CLas-induced hormonal perturbation reshapes the microbiome, while the remodeled microbiome further modifies host-derived metabolites. Such reciprocal interactions may shift the citrus holobiont from a homeostatic metabolic state toward a disease-associated state characterized by altered hormone metabolism, microbial community structure, and metabolite profiles. Within this framework, dehydrovomifoliol may serve as a molecular signature and potential mediator of the transition between holobiont states. Its accumulation is consistent with enhanced microbial conversion of host-derived ABA, while its reported biological activities raise the possibility that it may further influence host cellular processes or plant-microbe interactions (Kyaw et al., 2022; Gyi et al., 2025). Whether dehydrovomifoliol actively contributes to HLB development or primarily reflects the metabolic consequences of infection remains unresolved. Nevertheless, its formation provides a mechanistic example of how a host-derived phytohormone can be incorporated into microbial metabolism and returned to the host environment as a chemically distinct metabolite. Taken together, our findings support a model in which CLas infection reorganizes the host-microbiome metabolic network rather than simply perturbing host hormone levels. The resulting metabolic rewiring may alter chemical communication between citrus and its associated microorganisms and contribute to the establishment of a disease-associated holobiont state. If metabolites generated through such host-microbial transformations subsequently influence immune signaling, HLB may represent a disease state associated with disruption of holobiont metabolic and immune homeostasis, rather than dysfunction of the plant immune system alone. This framework extends the role of microbial hormone metabolism from a mechanism of pathogen adaptation to a potential determinant of the physiological state of the entire plant holobiont.

## 5 Conclusion

Our study demonstrates that CLas infection is associated with a marked shift in host hormone homeostasis, characterized by reduced ABA and increased dehydrovomifoliol levels in citrus tissues. We show that CLas and CLas-associated *Flavobacterium* spp. metabolize ABA into dehydrovomifoliol and that ABA promotes bacterial growth *in vitro*. These findings suggest that pathogen-associated microbial activity contributes to hormone imbalance during infection through ABA degradation. Moreover, the accumulation of dehydrovomifoliol, a bioactive compound, highlights a previously unrecognized link between pathogen-driven metabolic reprogramming and the generation of secondary metabolites in HLB citrus. Together, this work provides insight into the role of microbial metabolism in shaping host physiology and suggests new perspectives on the functional consequences of plant-pathogen-microbiome interactions.

## Data Availability

The original contributions presented in the study are included in the article/Supplementary material, further inquiries can be directed to the corresponding author/s.
