# Supplementary material for: Abscisic acid metabolism to dehydrovomifoliol by Candidatus Liberibacter asiaticus and CLas-associated Flavobacterium strains CGRL1 and CGRL2
Source: Front Microbiol. 2026 Sep 15;17:1940325. doi: 10.3389/fmicb.2026.1940325 (PMC13619911; doi:10.3389/fmicb.2026.1940325)
Supplement: Supplementary file 1 [file Data_Sheet_1.PDF]

Supplementary Material

Abscisic Acid Metabolism to Dehydrovomifoliol by *Candidatus* Liberibacter asiaticus and CLas-Associated *Flavobacterium* Strains CGRL1 and CGRL2

Kosuke Yamada, Azusa Fukushima, Takashi Fujikawa, Hiroyuki Nakagawa, Kosuke Namba, Kazuki Fujiwara[*]

*** Correspondence:** Kazuki Fujiwara, fujikazu@meijo-u.ac.jp

Supplementary Table S1: LC-MS/MS parameters optimized for the detection of abscisic acid and related compounds.

Analyte	Q1 precursor ion (*m/z*)	Q3 product ion (*m/z*)	Declustering Potential (DP)	Entrance potential (EP)	Collision Energy (CE)	Collision cell exit potential (CXP)
(*Z*)-ABA	263.069	152.8	-60	-10	-16	-25
	263.069	152.4	-60	-10	-18	-9
	263.069	219.0	-60	-10	-20	-17
(*E*)-ABA	263.069	219.0	-60	-10	-20	-17
	263.069	204.0	-60	-10	-22	-11
	263.069	201.0	-60	-10	-22	-11
Dehydrovomifoliol	220.954	205.6	-65	-10	-28	-13
	220.954	178.7	-65	-10	-18	-15
	221.004	205.7	-70	-10	-26	-15

Supplementary Table S2: Chemical composition of mineral salt medium.

Component	Manufacturer	Quantity (/L)	Reference
Disodium Hydrogen phosphate	Yoneyama	5.55 g	Yuzikhin et al., (2021)
Potassium Dihydrogen Phosphate	Kanto	3.00 g	
Ammonium Chloride	Kanto	1.00 g	
Magnesium Sulfate Heptahydrate	Wako	0.61 g	
Calcium Chloride Dihydrate	Wako	0.13 g	
Glucose	Wako	2.00 g	This study[a]

a. The medium composition was modified by additional glucose.
